# Longitudinal relations between symptoms, neurocognition, and self-concept in schizophrenia

**DOI:** 10.3389/fpsyg.2015.00917

**Published:** 2015-07-03

**Authors:** Klaus Hesse, Levente Kriston, Andreas Wittorf, Jutta Herrlich, Wolfgang Wölwer, Stefan Klingberg

**Affiliations:** ^1^Department of Psychiatry and Psychotherapy, University of TübingenTübingen, Germany; ^2^Department of Medical Psychology, University Medical Center Hamburg-EppendorfHamburg, Germany; ^3^Department of Psychiatry and Psychotherapy, University of FrankfurtFrankfurt, Germany; ^4^Department of Psychiatry and Psychotherapy, Medical Faculty, University of DuesseldorfDuesseldorf, Germany

**Keywords:** cognitive models, structural equation modeling, self-esteem, psychological model, self-schema

## Abstract

**Objective:** Cognitive models suggest that the self-concept of persons with psychosis can be fundamentally affected. Self-concepts were found to be related to different symptom domains when measured concurrently. Longitudinal investigations to disentangle the possible causal associations are rare.

**Method:** We examined a sample of 160 people with a diagnosis of schizophrenia who took part in a psychotherapy study. All participants had the DSM-IV diagnosis of a schizophrenia and pronounced negative symptoms. Neurocognition, symptoms, and self-concepts were assessed at two time points 12 months apart. Structural equation modeling was used to test whether symptoms influence self-concepts (scar-model) or self-concepts affect symptoms (vulnerability model).

**Results:** Negative symptoms correlated concurrently with self-concepts. Neurocognitive deficits are associated with more negative self-concepts 12 months later. Interpersonal self-concepts were found to be relevant for paranoia.

**Conclusion:** The findings implicate that if deficits in neurocognition are present, fostering a positive self-concept should be an issue in therapy. Negative interpersonal self-concept indicates an increased risk for paranoid delusions in the course of 1 year. New aspects for cognitive models in schizophrenia and clinical implications are discussed.

## Introduction

In cognitive models of paranoid delusions and negative symptoms negative self-concepts in terms of reduced self-efficacy, self-acceptance, self-esteem, and expectancies for pleasure play a major role (Rector et al., 2005; Kesting and Lincoln, 2013). Self-concepts integrate cognitive, emotional, and motivational reflections of the self. For the emotional aspect, self-esteem, as an evaluative self-concept, is a prominent factor as well as an important outcome in mental health research. Self-concepts are central to the health care of people with schizophrenic psychoses as a core element of quality of life (Weinberg et al., 2012) as well as a potential mediator between treatment and outcome. Self-esteem in schizophrenia was found to be linked to depression (Cavelti et al., 2012a), quality of life (Staring et al., 2009), functional outcomes (Vracotas et al., 2012), negative symptoms (Palmier-Claus et al., 2011a), and positive symptoms (Barrowclough et al., 2003; Thewissen et al., 2011).

**FIGURE 1:**
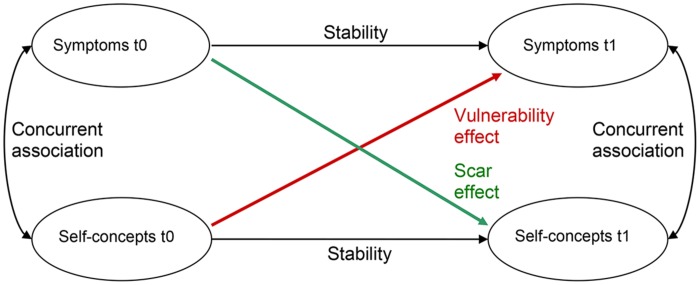
**Theoretical model integrating the scar and the vulnerability model at two time points (t0 and t1); adapted from Sowislo and Orth (2013)**.

The relationship between symptoms and self-concepts are typically discussed in two ways (Klein et al., 2011). First, negative self-concepts can be regarded as vulnerability for higher symptom severity as the capability for coping with stressful events might be reduced (Zubin and Spring, 1977; Bentall et al., 1994). Second, symptoms might induce negative changes in the self-concepts, which may be considered as a scar (Lewinsohn et al., 1981). The ideas of the vulnerability and the scar model are summarized graphically in **Figure 1**. In a meta-analysis of 77 studies with representative, non-representative, and clinical samples the vulnerability model showed stronger effects than the scar model for depression (Sowislo and Orth, 2013).

Although the course of self-concepts and symptoms has been studied extensively in depressive disorders, evidence is scarce in psychotic disorders. Most available studies refer to the development and course of paranoia. Cognitive models state that dysfunctional self-concepts contribute to paranoid delusions (Bentall et al., 1994; Garety et al., 2001; Freeman et al., 2002). Recent clinical (Thewissen et al., 2011), non-clinical (Thewissen et al., 2008), and experimental evidence (Palmier-Claus et al., 2011b; Kesting et al., 2013) elucidated the association between self-concepts and positive symptoms, especially paranoid delusions. Particularly the relationship between self-esteem, stigma, insight, depression, and positive symptoms has been studied comprehensively (Lysaker et al., 2007; Cavelti et al., 2012b; Erickson and Lysaker, 2012). In their review regarding this topic, Kesting and Lincoln (2013) concluded that negative interpersonal self-concepts and low self-esteem can lead to persecutory delusions.

Neurocognition is a reliable predictor of functional outcome (Green et al., 2000; Bowie et al., 2010). These cognitive dysfunctions are relative stable through the course of the illness and are merely unaffected by medication (Harvey and Keefe, 2001). Neurocognitive deficits are even present in first-episode populations (Reichenberg et al., 2009). Verbal memory performance is enhanced in the year after remission of positive symptoms but performance levels remain impaired (Wittorf et al., 2004). The same picture is shown for high-risk patients; the deficits are viable before onset, but improve over time (Bora and Murray, 2014). Intensified programs of cognitive remediation can yield to better cognitive performance and functioning (Wykes et al., 2011; Sanchez et al., 2014).

In the cognitive model of negative symptoms, defeatist beliefs are related to symptoms like avolition, anhedonia, and affective flattening (Rector et al., 2005). Some studies confirmed these associations between self-reported expectancies about competences, success, or acceptance, and observer-rated negative symptoms (Grant and Beck, 2009). In the same study the authors found that defeatist beliefs about oneself mediate the association between neurocognition and functional outcome, supporting the scar model for neurocognition. Furthermore, interpersonal self-concepts and self-esteem correlated with negative symptoms (Lincoln et al., 2011). Palmier-Claus et al. (2011b) reported data supporting the vulnerability model for negative symptoms in early psychosis. In their study, the change in self-concepts predicted the course of negative symptoms.

Self-concepts can play a substantial role for subjective well being and for recovery. Especially self-esteem and self-efficacy have been pointed out as important personal traits within the recovery process (Yanos and Moos, 2007). Self-concepts are targets in narrative enhancement therapy (Yanos et al., 2011), schema-therapy (Bortolon et al., 2013), meta cognitive therapy (Moritz et al., 2014), and acceptance, and commitment therapy (Gaudiano and Herbert, 2006). Especially in the narrative enhancement therapy fragmented self-narratives and self-stigma are targeted. These approaches could enrich cognitive behavioral therapy for psychosis (Tai and Turkington, 2009) as well as self-concepts could give a new focus for family interventions (Hesse et al., 2015; Yesufu-Udechuku et al., 2015).

In the present study, our purpose was to examine the plausibility of the scar and vulnerability models regarding the clinically most significant areas of symptoms and neurocognition in people with schizophrenic psychosis. First, we expected that all symptom domains, including neurocognitive deficits, are associated with self-concepts. Second, we expected to find further evidence for the vulnerability effect referring to positive and negative symptoms and a scar effect referring to neurocognition.

## Materials and Methods

### Subjects and Procedures

The original sample comprised 198 outpatients who participated in a randomized controlled trial for the treatment of negative symptoms with cognitive behavioral therapy in three University Hospitals (TONES-study, ISRCTN25455020; Klingberg et al., 2009, 2011). All participants gave written informed consent. The study was conducted in accordance with the Declaration of Helsinki and the guidelines of the local University ethics committees (Tuebingen, Frankfurt, and Duesseldorf). The DSM-IV diagnosis of schizophrenia was confirmed by a structured clinical interview (SCID-I). Assessment of symptoms was performed by trained raters. The inclusion and exclusion criteria are reported in detail in the study protocol (Klingberg et al., 2009). Study participants had to have at least moderate negative symptoms and no severe depressive (PANNS G07, depression ≥6) or severe positive symptoms [any item of the standard PANSS positive scale (P1, P2, P3, P4, P5, P6, P7) ≥6]. The study population represents a more homogenous subgroup of people diagnosed with schizophrenia, than a random or unselected sample. A little loss of data (19%) occurred due to reward given for ratings to all participants and external data monitoring. We analyzed data of 160 participants whose follow-up data (12 months) were available. Missing data were imputed with expectation-maximization imputation models.

### Measures and Latent Variable Construction

We grouped indicators to five latent constructs and tested the measurement adequacy empirically (Klingberg et al., 2006; Nuechterlein et al., 2008; Kim et al., 2012; Khan et al., 2013). As indicators of a latent construct may differ in the degree to which they represent the latent construct, we examined factor loadings as a measure of the strength of association between the indicator and the construct.

*Negative symptoms* were measured by the *Positive and Negative Symptom Scale (PANSS) and the Scale for the Assessment of Negative Symptoms (SANS).* The corresponding factor loadings to the negative symptoms factors in our analyses can be considered as high (0.86–0.96), respectively.

*Paranoia* was measured by the “delusions” item from the PANSS (item P1) and the “vsuspiciousness/persecution” item from the PANSS (item P6). The factor loadings ranged from 0.51 to 0.84.

Two domains of *neurocognition*, verbal recall and processing speed, were selected as particularly relevant. The *Trail Making Test (TMT)* consists of two parts, one (part A) measures mainly processing speed. Verbal memory was measured by the *Verbaler Lern und Merkfähigkeitstest Test (VLMT).* The two tests represent two different domains of neurocognitive functioning, therefore lower factor loadings were expected. For sake of content validity of the factor we decided to keep verbal memory in the construct. The factor loadings of the tests ranged from 0.41 to 0.81, indicating that verbal memory is not as well represented in the neurocognitive functioning construct as processing speed.

*Self-concepts* were assessed with the *Frankfurt Self-Concept Scales* (FSKN; Deusinger, 1986). This inventory comprises 10 one-dimensional scales with specific self-concepts concerning relevant aspects of the self. The internal consistency of the scales was highly satisfactory (α = 0.93–0.97; *n* = 1794). The questionnaire has been used in psychosis research frequently (Lincoln et al., 2010, 2011; Wittorf et al., 2010). We used six subscales for our analysis, three to measure positive self-concept, and three to measure interpersonal self-concept.

The self-concepts “general achievement,” “solving daily problems,” and “self-esteem” were used to measure *positive self-concept*. The factor loadings of these subscales ranged from 0.88 to 0.93. *Interpersonal self-concept* was measured with three subscales from the FSKN: “valued by others,” “ability to make contact with other people “and” emotions and relations to others.” The factor loadings of these subscales ranged from 0.59 to 0.86.

### Statistical Analysis

First we checked if the two psychotherapeutic interventions to which patients were allocated in the RCT have any significant differential treatment effect on the variables of interest. Analysis of covariance (ANCOVA) was conducted with t1 as the dependent and t0 and treatment group as independent variables for each symptom and self-concept. We used structural equation modeling (SEM) techniques to test the main hypotheses of longitudinal associations. SEM is a confirmative technique allowing the construction of latent variables by observed indicators and testing the relations between the latent constructs.

We examined the vulnerability model and the scar model for different symptoms and self-concepts comparing estimates of strength of association and fit indexes. In a preparatory investigation of assumptions of SEM both skewness and kurtosis of the modeled indicators were within acceptable limits (Kline, 2011). A total of six longitudinal models were defined using data from baseline (t0) and 12-months follow-up (t1) with combinations of the two areas of self-concepts (positive self-concept and interpersonal self-concept) and three symptom domains (paranoia, negative symptoms, and neurocognition). We allowed autocorrelations between indicators over time. When Heywood cases (negative error variances) occurred, problematic autocorrelations have been fixed at 0. For each self-concept-symptom pair, an unrestricted model including all paths and thus allowing for both scar and vulnerability effects was estimated. Subsequently, partly restricted models omitting one path each representing the vulnerability or the scar model were fit, respectively. Finally, a fully restricted model excluding both scar and vulnerability effects was estimated.

All analyses were performed with AMOS and SPSS (Version 21.0. Armonk, NY: IBM Corporation).

## Results

The ANCOVAs for differential effects of the interventions resulted in no significant (*P* < 0.05) difference between the two groups for any reported variable. Therefore the treatment group was not considered in further analyses. Anyway, we report in the appendix on models incorporating the group factor to rule out influence of treatment.

The mean scores and SD for all variables are summarized in **Table 1**. The sample comprised only a few first-episode patients, and the majority was male. Level of occupation and of general functioning indicates that the sample was moderately to severely impaired. The sample is characterized by rather weak positive symptoms and moderate to severe negative symptoms. The mean results in the VLMT are about one SD lower than the results in an age-matched normative sample (*M* = 52.27, SD = 7.84; Lux et al., 1999). The time needed to complete the TMT A is more than one SD above the mean in the age-matched normative sample (*M* = 28.54, SD = 10.09; Tombaugh, 2004).

**Table 1:** Demographic and clinical characteristics of the sample.

	t0 (Baseline)		t1 (12 months)
	Frequency	Percent		
Female	66	41		
High school	84	52		
Occupation	44	28		
Married/with partner	65	41		
Adverse child events	31	19		
First episode	11	7		

	**Mean**	**SD**	**Mean**	**SD**

Age (years)	36.90	9.83		
Age at first psychiatric symptom (years)	23.77	8.74		
GAF (score)	59.58	8.91	63.34	11.47
Verbal IQ	108.72	16.72		
PANSS				
PANSS P01 (item score)	1.89	1.00	2.06	1.23
PANSS P06 (item score)	2.00	0.91	1.94	1.07
PANSS MNS (mean item score)	3.02	0.80	2.58	0.90
SANS (mean score)	2.01	0.65	1.69	0.78
TMT A (section)	38.68	15.71	33.59	14.11
VLMT learning (sum words)	45.16	10.66	46.69	11.79
FSKN				
General achievement (FSGA)	3.51	0.90	3.70	0.90
Solving daily problems (FSSP)	3.65	0.81	3.78	0.82
Self-esteem (FSSE)	3.68	1.05	3.95	0.99
Valued by others (FSVO)	3.69	0.99	3.87	1.03
Ability to make contact (FSAC)	3.73	0.84	3.91	0.78
Emotions and relationships (FSEO)	3.80	0.82	3.75	0.78

*N = 160; Occupation, Fulltime occupation or education; GAF, Global Assessment of Functioning, PANSS P01, delusions; P06, suspiciousness/persecution; MNS, modified negative symptom scale; SANS, Scale for the Assessment of Negative Symptoms; TMT A, Trail Making Test Trail A; VLMT, Verbaler Lern und Merkfähigkeitstest; FSKN, Frankfurt Self-Concept Scales*.

Negative symptoms were the first domain to be tested with regard to the vulnerability and the scar model. The fit of all models is good. The restricted model, omitting the paths representing the vulnerability and the scar model, does not significantly impact the model fit. The goodness of fit statistics are summarized in **Table 2**. Negative symptoms were fairly stable over time as indicated by a standardized coefficient of 0.66. Whereas the correlation between negative symptoms and positive self-concept was -0.32 at t0, it increased marginally to -0.44 at t1. The test of models with negative symptoms and interpersonal self-concepts result in similar results as shown in **Table 2**. Incorporating treatment group in the model (see Supplementary Figure S1), did not change these results. In summary, for negative symptoms our data did not support either the scar or the vulnerability model.

**Table 2:** Goodness-of-fit indices of the tested models and model comparisons.

	Chi-sq	Chi-sq/*df*	CFI	TLI	RMSEA	BIC	AIC	Coefficient (SE; *P*)
Threshold for good models	n.a.	≤2	≥0.950	≥0.950	≤0.050	l.v.p.	l.v.p.	*P* < 0.05
**Negative Symptoms**
**Positive self-concept**								
Unrestricted model (*df* = 26)	22.05; *P* = 0.63	0.882	1.000	1.004	0.000	174.31	82.05	
Scar model (*df* = 27)	22.18; *P* = 0.68	0.853	1.000	1.005	0.000	169.36	80.18	-0.10 (0.07; 0.128)
Vulnerability model (*df* = 27)	24.35; *P* = 0.56	0.937	1.000	1.002	0.000	171.53	82.35	0.02 (0.08; 0.719)
Restricted model (*df* = 28)	24.39; *P* = 0.61	0.903	1.000	1.003	0.000	166.50	80.39	
**Interpersonal self-concept**								
Unrestricted model (*df* = 26)	25.78; *P* = 0.42	1.031	0.999	0.999	0.014	178.03	85.78	
Scar model (*df* = 27)	27.17; *P* = 0.40	1.045	0.999	0.998	0.017	174.35	85.17	-0.08 (0.08; 0.232)
Vulnerability model (*df* = 27)	25.78; *P* = 0.48	0.992	1.000	1.000	0.000	172.96	83.78	0.01 (0.08; 0.943)
Restricted model (*df* = 28)	27.18; *P* = 0.45	1.007	1.000	1.000	0.006	169.28	83.18	
**Neurocognition**
**Positive self-concept**								
Unrestricted model (*df* = 26)	27.54; *P* = 0.28	1.145	0.997	0.995	0.030	184.87	89.54	
Scar model (*df* = 27)	27.90; *P* = 0.31	1.116	0.998	0.996	0.027	180.16	87.90	0.26 (0.02; 0.008)
Vulnerability model (*df* = 27)	38.00; *P* = 0.05	1.520	0.989	0.981	0.057	190.25	97.99	-0.05 (0.63; 0.563)
Restricted model (*df* = 28)	38.14; *P* = 0.06	1.467	0.990	0.983	0.054	185.32	96.14	
**Interpersonal self-concept**								
Unrestricted model (*df* = 26)	25.63; *P* = 0.37	1.125	0.998	0.996	0.020	183.91	87.63	
Scar model (*df* = 27)	26.43; *P* = 0.39	1.096	0.998	0.997	0.019	179.61	86.43	0.25 (0.02; 0.02)
Vulnerability model (*df* = 27)	31.54; *P* = 0.17	1.218	0.992	0.985	0.040	184.72	91.54	-0.07 (0.80; 0.39)
Restricted model (*df* = 28)	32.27; *P* = 0.19	1.195	0.992	0.987	0.038	180.31	90.24	
**Paranoia**
**Positive self-concept**								
Unrestricted model (*df* = 26)	28.57; *P* = 0.24	1.191	0.996	0.992	0.035	185.90	90.57	
Scar model (*df* = 27)	31.21; *P* = 0.18	1.248	0.994	0.990	0.040	183.46	91.20	0.03 (0.07; 0.714)
Vulnerability model (*df* = 27)	28.70; *P* = 0.28	1.148	0.997	0.994	0.030	180.95	88.70	-0.18 (0.15; 0.097)
Restricted model (*df* = 28)	31.30; *P* = 0.22	1.204	0.995	0.992	0.036	178.48	89.30	
**Interpersonal self-concept**								
Unrestricted model (*df* = 26)	29.41; *P* = 0.21	1.225	0.992	0.985	0.038	186.74	91.41	
Scar model (*df* = 27)	34.02; *P* = 0.11	1.361	0.987	0.976	0.048	186.28	94.02	0.01 (0.08; 0.904)
Vulnerability model (*df* = 27)	29.42; *P* = 0.25	1.117	0.994	0.988	0.033	181.68	89.42	-0.25 (0.14; 0.029)
Restricted model (*df* = 28)	34.03; *P* = 0.13	1.309	0.988	0.980	0.044	181.21	92.03	

*Chi-sq, discrepancy chi-squared statistic; df, degrees of freedom; Chi-sq/df, normed chi-squared statistic; Coefficient, the standardized estimates in the unrestricted model; SE, Standard error of the coefficient; *P*, significance level of the coefficient; CFI, Comparative Fit Index; TLI, Tucker-Lewis Index; RMSEA, Root Mean Squared Error of Approximation; BIC, Bayes Information Criterion; l.v.p., lower values preferred (only for model comparisons)*.

**FIGURE 2:**
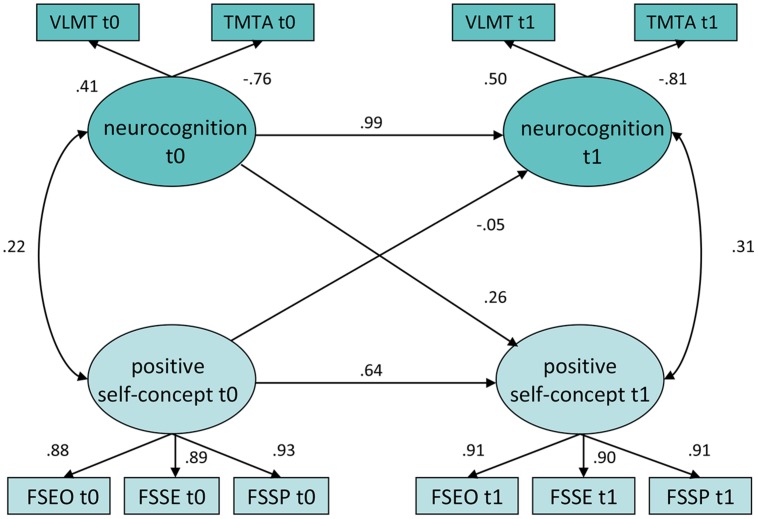
**Unrestricted longitudinal model of Positive Self-concept and Neurocognition**. Rectangles indicate observed indicator variables. Ovals indicate unobserved latent variables. Figures on single-headed arrows indicate standardized regression weights; figures on double-headed arrows correlations. Error variables are omitted. TMT A, Trail Making Test Trail A; (VLMT) Verbaler Lern und Merkfähigkeitstest; Frankfurt Self-Concept Scales: (FSGA, general achievement; FSSP, solving daily problems; FSSE, self-esteem). The overall model fit was χ^2^ 27.538, *df* = 24, *P* < 0.280; CFI = 0.997, TLI = 0.995, RMSEA = 0.030 (0.000 – 0.074).

With regard to neurocognition, the scar model was identified as the best model both for positive and interpersonal self-concepts. These results are presented in **Table 2** and **Figure 2**. Neurocognition is highly stable over time indicated by a high auto-regression coefficient. The standardized coefficient from neurocognition at baseline to positive self-concept at follow-up representing the scar model is 0.26 with *p* = 0.008. In nested model comparison for positive self-concept, the difference between the restricted model and the scar model is significant, indicating a substantially increased model fit for the scar model than for the restricted model (*df* = 1; ΔX^2^ = 10.24; *p* = 0.001). For interpersonal self-concepts the same pattern is depicted; the scar path coefficient (0.25; *p* > 0.019) and the difference to the restricted model are both significant (*df* = 1; ΔX^2^ = 5.80; *p* = 0.016). When treatment group is added to the model (see Supplementary Figure S2), the relationship between neurocognition at t0 and positive self-concept in t1 remains significant (*p* = 0.008). In summary, for neurocognition the data supported the scar hypothesis.

**FIGURE 3:**
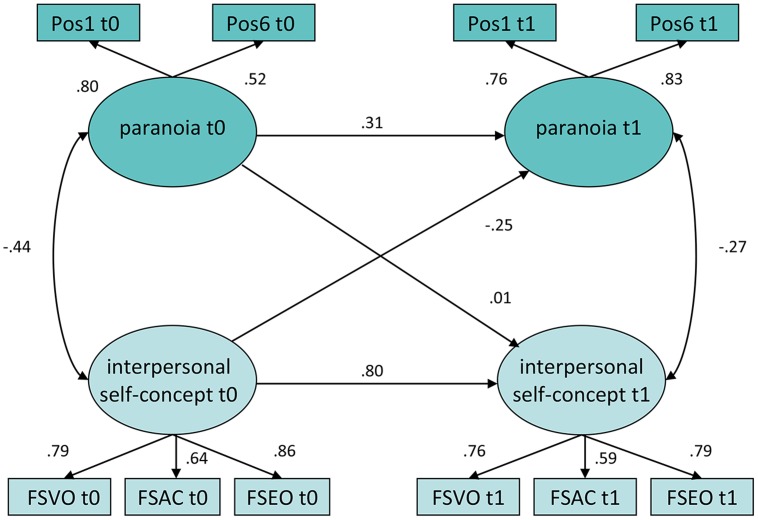
**Unrestricted longitudinal model of interpersonal self-concept and paranoia.** Rectangles indicate observed indicator variables. Ovals indicate unobserved latent variables. Figures on single-headed arrows indicate standardized regression weights; figures on double-headed arrows correlations. Error variables are omitted. Pos1, PANNS P01 delusions; Pos06, PANSS P6 suspiciousness/persecution; Frankfurt Self-Concept Scales: (FSVO, valued by others; FSAC, ability to make contact with other people, FSEO, emotions and relations to others). The overall model fit was χ^2^ = 29.41, *df* = 24, *P* = 0.21; *CFI* = 0.992, *TLI* = 0.985, *RMSEA* = 0.038 (0.000 – 0.078).

For paranoia, the vulnerability model showed better fitting indices. The unrestricted model for interpersonal self-concepts is presented in **Figure 3**. Whereas the concurrent correlation between paranoia and self-concepts is -0.44 at baseline, it decreases to -0.27 12 months later. The stability of paranoia is smaller than for negative symptoms or neurocognition with a standardized coefficient of 0.31. The standardized coefficient from interpersonal self-concept at baseline to paranoia at follow-up representing the vulnerability model is -0.25 with *p* < 0.029. Moreover the chi-square statistics of the vulnerability model fits significantly superior than the restricted model (*df* = 1; ΔX^2^ = 4.60; *p* = 0.032). Although the results were fairly comparable for the models with positive self-concept, the coefficient representing the vulnerability model did not reach the threshold for strict statistical significance (*p* < 0.097). As well, the chi-square statistics between the vulnerability model and the restricted model did not differ significantly, indicating no significant incremental fit for the vulnerability model with positive self-concepts.(*df* = 1; X^2^ = 2.17; *p* = 0.141). When treatment group is added to the model (see Supplementary Figure S3), the relationship between paranoid delusions at t0 and interpersonal self-concept in t1 remains significant (*p* = 0.031). In summary, for paranoia the data supported the vulnerability model, particularly with regard to interpersonal self-concept.

## Discussion

Cognitive models on negative symptoms, positive symptoms, and neurocognition can inform treatment development as they shed light on the development and maintenance of symptoms (Garety et al., 2001, 2007; Freeman et al., 2002; Rector et al., 2005; Kesting and Lincoln, 2013). In order to obtain a robust evidence base, these models need to be tested by different methodologies including epidemiological studies (Krabbendam et al., 2002; Fowler et al., 2006), experimental data with healthy controls, or clinical samples (Kesting et al., 2013), as well as longitudinal data from clinical samples like the study presented in this article.

### Negative Symptoms

Cognitive models of *negative symptoms* (Rector et al., 2005) as well as the psychotherapeutic rationale (Staring et al., 2013) rely on defeatist beliefs and negative self-concepts. Most studies use measures of defeatist attitudes and expectancies such as measured by the dysfunctional attitude scale (Beck et al., 2013). In our study, negative symptoms were associated concurrently with self-concepts as predicted by the cognitive model of negative symptoms. Contrary to our expectation, self-concept at pre-treatment did not predict negative symptoms after 12 months. The construct of negative symptoms has been stable, thus there was change in individuals and in the mean, but relative low change in the individual residuals. However, the longitudinal analysis did not support an influence, like found in first-episode patients (Palmier-Claus et al., 2011a). As in our study the time between t0 and t1 was 12 months, multiple causation might have influenced negative symptoms as well as self-concepts during this period. The variance in our sample is limited due to the inclusion criteria; this might have limited the covariances as well. For further research a shorter duration of measurement intervals is supposed to test.

### Neurocognition

As hypothesized, neurocognitive functioning at baseline predicted positive self-concept after 12 months. Yet, self-concepts are not in the focus of interest in research on *neurocognition* in people with psychosis. In the general population there is strong evidence that people can estimate their cognitive abilities well (Freund and Kasten, 2012). Our findings support the scar model. Possibly service-users perceive the loss of memory function and processing speed during the course of the disorder and integrate them in a negative self-concept. In concurrent analyses of people with schizophrenia, defeatist beliefs operated as a mediator between neurocognitive impairments and negative symptoms (Grant and Beck, 2009). These results demonstrate the importance of functional illness-concepts. In a cross-sectional model of visual perception, social cognition, and social functioning the same mediating effect of negative beliefs about the self was found (Green et al., 2012). It is plausible that more negative self-concepts may lead to negative symptoms due to the perception of neurocognitive deficits and maladaptive illness-concepts.

### Paranoid Delusions

In our study, evidence was found for a prediction of paranoia after 12 months by interpersonal self-concept at pre-treatment. Other researchers yielded empirical support for the vulnerability model in *paranoid delusions* was as well (Fowler et al., 2012; Kesting and Lincoln, 2013). For example, in daily life reports of fluctuations in self-esteem predicted the development of paranoia (Thewissen et al., 2008). Some cross-sectional studies found positive correlations between self-concepts and positive symptoms (Barrowclough et al., 2003) or paranoid delusions (Smith et al., 2006). In the data presented above the stability of paranoid delusions was weak, primarily indicating that most people involved in this trial had only modest paranoid delusions at entry but some of them relapsed in the course of the study. The scales “negative self” and “negative others” of the Brief Core Schema Scale (BCSS; Fowler et al., 2006) have shown positive correlations with paranoid delusions (Freeman et al., 2013; Garety et al., 2013). There is a slight difference between the “negative others” scale in the BCSS and the interpersonal self-concepts measured in the FSKN. Whereas the BCSS assesses appraised threat from others, the items used in our study are formulated as self-concepts, i.e., how the person is thinking about itself in social relationships. The three scales which has been used to measure interpersonal self-concepts reflect the feelings of being valued by others, trustworthy for others and competent in making contacts. In the BCSS one item is for instance: “Other people are supportive,” whereas in the FSKN a corresponding inverted item is “With many of my friends, I’m afraid that when I need them they won’t be there for me.” In our study, the more global positive self-concepts did not support the vulnerability model; the path from positive self-concept at baseline to paranoia at follow-up did not reach statistical significance. The long interval of 12 months, the limited variance in paranoia and the sample size may have caused these non-significant findings. Lincoln et al. (2010) found although that paranoia was not associated with self-esteem but with interpersonal self-concepts. In our study, interpersonal self-concepts predicted paranoia too, hence when psychological models of paranoia are studied, interpersonal self-concepts in addition to more general positive, or negative self-concepts should be considered. Bentall et al. (2001) have hypothesized that people with tendencies to paranoid delusions avoid negative beliefs about the self, by attributing threatening events to other persons. Interpersonal self-concepts could reflect not only the self though how we see ourselves in social context and how we see other people in relation to us. Our findings support the model of persecutory delusions of Garety et al. (2001) and Freeman et al. (2002) who proposed that certain beliefs about the self and others are important factors in the development of persecutory delusions.

### Clinical Implications

There may be some clinical implications for our findings, assuming that cognitive behavioral therapy for psychosis is an effective treatment, one mechanism of change could be the improvement of self-concepts. Interpersonal self-concepts could be influenced by the quality of the therapeutic alliance in psychotherapy, which is indeed a common effect in the treatment of schizophrenia (Frank and Gunderson, 1990) as well as in every therapeutic intervention (Martin et al., 2000). We can speculate that in many therapeutic settings interpersonal self-concepts are influenced as the therapeutic relationship might be a positive model in terms of trustworthiness, reliability, and acceptance. The possible change in the interpersonal self-concept due to the therapy could be one explanation for the reduction in positive symptoms in supportive therapies (Penn et al., 2004) and for symptom changes during therapy even when they are not directly addressed. When neurocognitive deficits are seen in people with schizophrenia, interventions aiming at compensating deficits and modifying dysfunctional attitudes and self-concepts could be helpful in reducing negative performance expectancies and negative symptoms. When neurocognitive deficits are present, minimizing the deficits is crucial and partly possible (Wykes et al., 2011). The awareness of cognitive impairments is negatively correlated with self-esteem (Cella et al., 2014), therefore when neurocognition does not remit, service-users should be helped in accepting and destigmatizing limits caused by symptoms. For this purpose psychological interventions could be helpful, like combinations of cognitive therapy, and cognitive remediation (Greenwood et al., 2005; Wykes et al., 2011). For this purpose cognitive intervention could focus more on interpersonal self-concepts and narrative enhancements (Yanos et al., 2011) to protect people with schizophrenia from relapse to paranoid delusions.

### Strengths and Limitations

A main strength of our study is that patients have been investigated and followed-up over a period of 12 months. From the 198 patients interviewed at baseline we had almost complete data from 160 participants 12 months later, indicating a low risk of bias due to informative censoring. Whereas other studies showed effects for some hours (Thewissen et al., 2008) up to 9 months (Fowler et al., 2012), in our analysis the interval was 12 months. The relatively small coefficients have to be interpreted in this context.

There are some limitations in the study. First, the study is part of randomized controlled trial with systematic therapy regime. We tried to rule out influences from treatment statistically; anyway a sample without explicit psychotherapy would be more adequate to test the hypotheses. Second, the tested models had to be simple, because the sample size of 160 participants limits more complex structural equation models (e.g., a single model including all tested constructs and associations simultaneously). Furthermore, our sample consists of patients with predominantly negative symptoms and relatively weak positive symptoms, and thus might limit the possibility of generalization. On the other hand a strength of the study is to include a relative large sample of people with distinct inclusion criteria and a relative homogenous phenotype. Neurocognition as measured in this study consisted only of verbal memory and processing speed, other important domains like executive functions or verbal fluency were not included. We had other measures in the dataset available, but we could not reach appropriate model fits when including measures of attention and problem solving. Nevertheless we included two good established markers for neurocognition in our analysis. The Hopkins Verbal Learning Test (a similar verbal learning test as we used) and the TMT are included in the MATRICS-Battery, both are correlated with functioning and have the highest ratings for practicability by experts (Nuechterlein et al., 2008).

## Conclusion

We found some evidence for the importance of self-concepts in the course of symptoms in people with schizophrenia. We could find evidence for the scar model in neurocognition: global positive self-concepts as well as interpersonal self-concepts seem to be endangered when neurocognitive impairments occur. This study provides further evidence for a vulnerability model of paranoia: the presence of a negative interpersonal self-concept is a risk factor for paranoid delusions. This result is consistent with theories proposing a relationship between negative social experiences, mood, self-concepts, and paranoia (Garety et al., 2001; Freeman, 2007; Kesting and Lincoln, 2013).

## Conflict of Interest Statement

The authors declare that the research was conducted in the absence of any commercial or financial relationships that could be construed as a potential conflict of interest.
