# Supplementary material for: Longitudinal relations between symptoms, neurocognition, and self-concept in schizophrenia
Source: Front Psychol. 2015 Jul 3;6:917. doi: 10.3389/fpsyg.2015.00917 (PMC4490211; doi:10.3389/fpsyg.2015.00917)
Supplement: Supplementary file 1 [file Image_1.PDF]

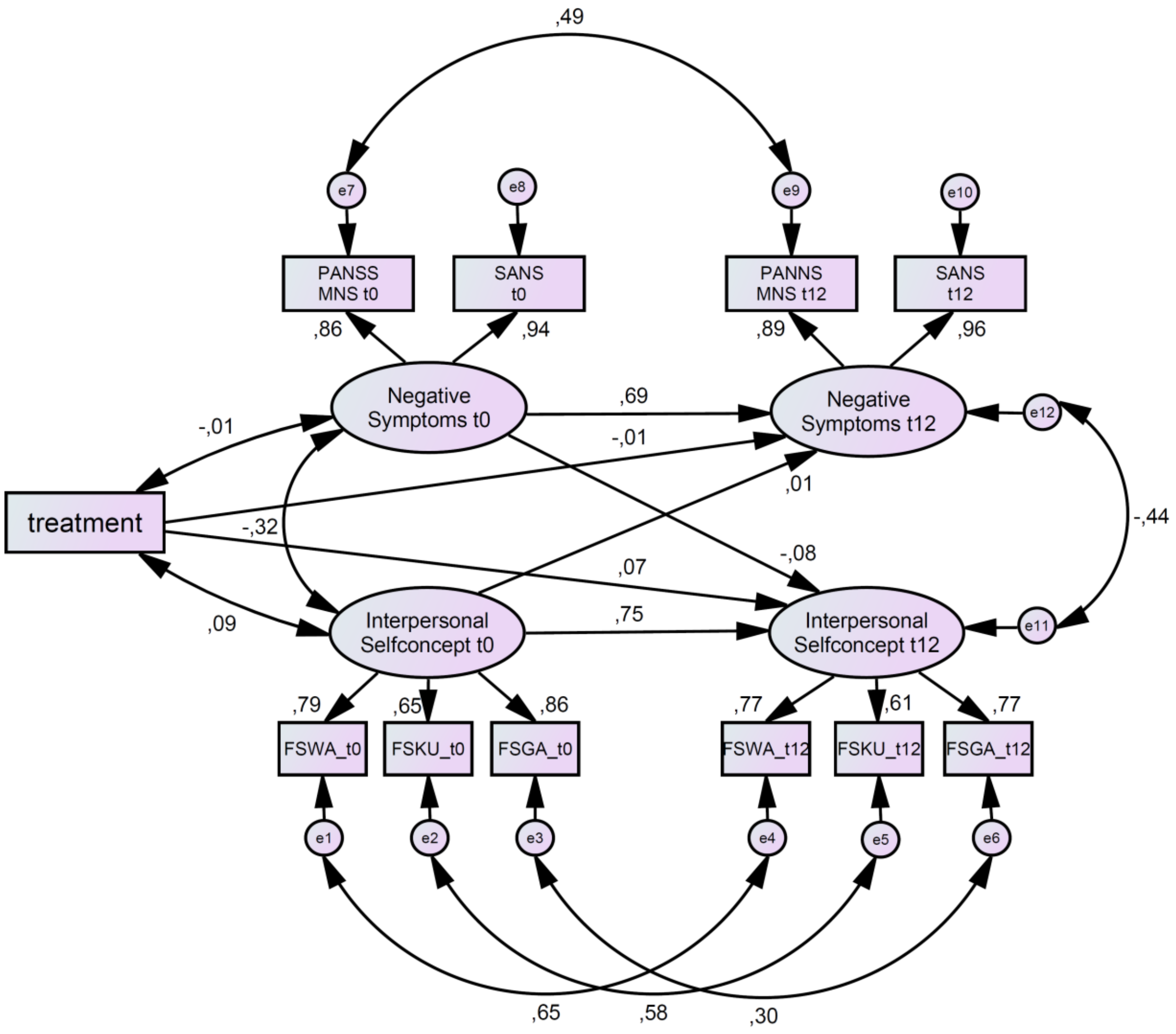

Supplementary Figure 1: Unrestricted longitudinal model of Positive Self-concept and Negative Symptoms, controlled for treatment

Rectangles indicate observed indicator variables. Ovals indicate unobserved latent variables. Figures on single-headed arrows indicate standardized regression weights; figures on double-headed arrows correlations. Error variables are indicated by circles.

Treatment: 0= cognitive remediation, 1= cognitive behavior therapy; PANSS MNS, PANSS modified negative syndrome scale; SANS, Scale for the Assessment of Negative Symptoms; Frankfurt Self-Concept Scales: (FSGA, general achievement; FSSP, solving daily problems; FSSE, self-esteem). The overall model fit was χ^2=31.39, df=30, P<0.397; CFI=0.999, TLI=0.998, RMSEA=0.017 (0.000 - 0.063).

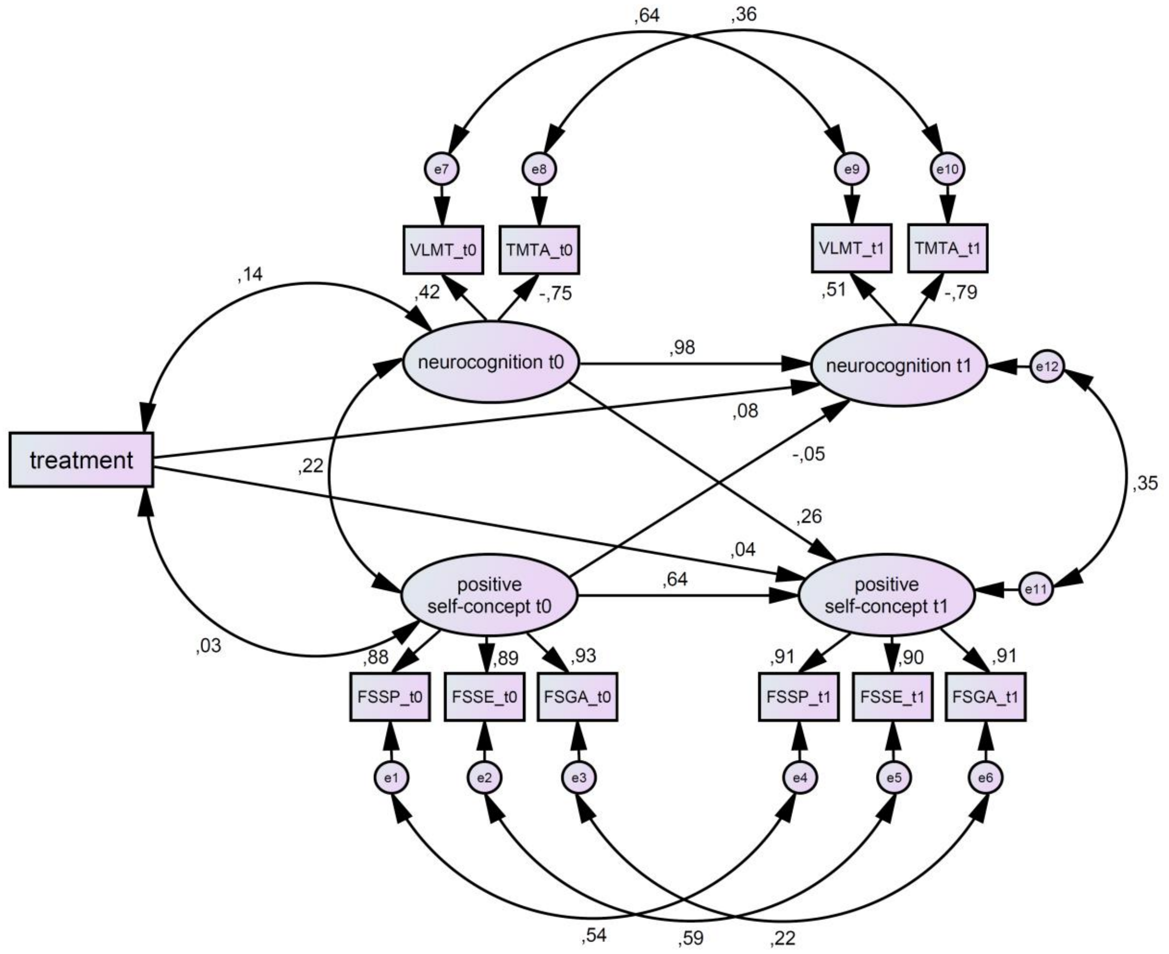

Supplementary Figure 2: Unrestricted longitudinal model of Positive Self-concept and Neurocognition, controlled for treatment.

Rectangles indicate observed indicator variables. Ovals indicate unobserved latent variables. Figures on single-headed arrows indicate standardized regression weights; figures on double-headed arrows correlations. Error variables are indicated by circles.

Treatment: 0= cognitive remediation, 1= cognitive behavior therapy; TMT A, Trail Making Test Trail A; VLMT, Verbaler Lern und Merkfähigkeitstest; Frankfurt Self-Concept Scales: (FSGA, general achievement; FSSP, solving daily problems; FSSE, self-esteem). The overall model fit was χ^2=31.39, df=30, P<0.397; CFI=0.999, TLI=0.998, RMSEA=0.017 (0.000 - 0.063).

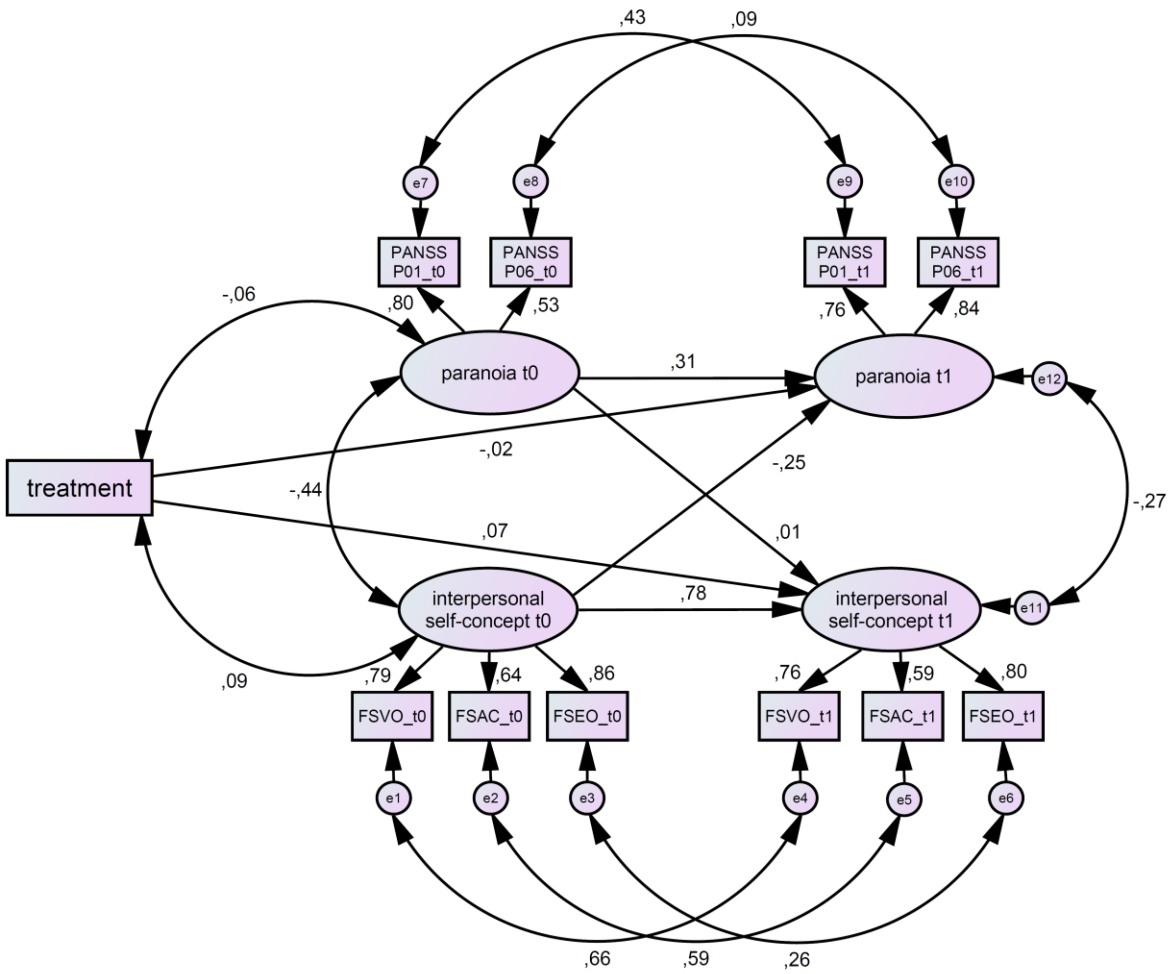

Supplementary Figure 3: Unrestricted longitudinal model of interpersonal self-concept and paranoia controlled for treatment.

Rectangles indicate observed indicator variables. Ovals indicate unobserved latent variables. Figures on single-headed arrows indicate standardized regression weights; figures on double-headed arrows correlations. Error variables are indicated by circles.

Treatment: 0= cognitive remediation, 1= cognitive behavior therapy PANNS P01, delusions; PANSS P06, suspiciousness/persecution; Frankfurt Self-Concept Scales: (FSVO, valued by others; FSAC, ability to make contact with other people, FSEO, emotions and relations to others). The overall model fit was χ^2=30.91, df=30, P=0.42; CFI=0.999, TLI=0.998, RMSEA=0.014 (0.000 - 0.062).
